# Alanine aminotransferase to aspartate aminotransferase ratio and hepatitis B virus on metabolic syndrome: a community-based study

**DOI:** 10.3389/fendo.2022.922312

**Published:** 2022-07-29

**Authors:** Ming-Shyan Lin, Huang-Shen Lin, Ming-Ling Chang, Ming-Horng Tsai, Yung-Yu Hsieh, Yu-Sheng Lin, Meng-Shu Tsai, Chia-Ling Yang, Mei-Yen Chen

**Affiliations:** ^1^ Division of Cardiology, Department of Internal Medicine, Chang Gung Memorial Hospital, Chiayi, Taiwan; ^2^ Graduate Institute of Clinical Medical Sciences, College of Medicine, Chang Gung University, Taoyuan, Taiwan; ^3^ Department of Nursing, Chang Gung University of Science and Technology, Chiayi, Taiwan; ^4^ Division of Hepatology, Department of Gastroenterology and Hepatology, Chang Gung Memorial Hospital, Taoyuan, Taiwan; ^5^ Department of Infection, Chang Gung Memorial Hospital, Chiayi, Taiwan; ^6^ Department of Pediatrics, Chang Gung Memorial Hospital, Yunlin, Taiwan; ^7^ Department of Gastroenterology and Hepatology, Chang Gung Memorial Hospital, Chiayi, Taiwan; ^8^ Department of Nursing, Chang Gung University, Taoyuan, Taiwan

**Keywords:** aminotransferase, community-based, education, metabolic syndrome, hepatitis B virus

## Abstract

**Background:**

The serum aminotransferase elevation in metabolic syndrome (MetS) reflected hepatosteatosis, but there is a conflict with the coexistence of viral hepatitis, especially for the hepatitis B virus (HBV). Thus, this study aimed to investigate the relationship between the alanine aminotransferase (ALT)/aspartate aminotransferase (AST) ratio, MetS, and HBV infection in a rural Taiwanese population.

**Methods:**

We conducted a cross-sectional analysis in southern Taiwan between March and December 2019. Multivariable logistic regression analyses adjusted for demographics, education, dietary behaviors, irregular exercise, substance use, and viral markers were performed to investigate the association between the ALT/AST ratio and MetS.

**Results:**

Altogether, 2,416 participants (891 men and 1,525 women; mean age, 64.1 ± 14.9 years) were enrolled. Of the participants, 22.7% (*n* = 519) were seropositive for viral hepatitis. In the multivariable analysis, age [odds ratio (OR) 1.02, 95% CI 1.01–1.03, *p *< 0.001], ALT/AST ratio >1 (OR 2.63, 95% CI 2.15–3.21, *p *< 0.001), education (OR 0.96, 95% CI 0.94–0.98, *p *< 0.001), and HBV seropositivity (OR 0.70, 95% CI 0.52–0.95, *p* = 0.021) were associated with the risk of MetS. The area under the curve of the ALT/AST ratio was 0.62 (95% CI 0.60–0.64, *p *< 0.001), and the cutoff value was >0.852 for the Youden index.

**Conclusion:**

An ALT/AST ratio >1 could be a simple index for MetS prediction during community checkups. In contrast to age and betel nut chewing, HBV seropositivity and higher education might be inversely associated with MetS. Aggressive health promotion for MetS prevention has emerged as essential in participants without HBV and with lower education levels. Further large-scale, longitudinal studies are needed to unlink these correlations.

## Introduction

Metabolic syndrome (MetS) is hypervalent (13.6% to 30.1%) in southern Taiwan (1), which is also a viral hepatitis endemic area. Twelve percent to 15% of the adult population infected with hepatitis B virus (HBV) remains hepatitis B surface antigen (HBsAg)-positive (2), and the prevalence of hepatitis C virus (HCV) seropositivity is remarkably high (overall 2%–4% in Taiwan). Although HCV independently increases the MetS burden (1, 3), the association between HBV and MetS is diverse (4–7). Central obesity and fatty liver have emerged as significant components of MetS, while steatohepatitis increases the mortality risk in the population (8, 9). Both viral hepatitis and fatty liver could induce abnormal alanine aminotransferase (ALT) and aspartate aminotransferase (AST) levels (3, 7), the data of which can be obtained from community checkups. Moreover, the predictive effect of aminotransferases on MetS may be influenced by viral hepatitis and lifestyle factors, including diet, exercise, and personal habits.

Serum aminotransferases increase along with being overweight [body mass index (BMI) ≥ 25 kg/m^2^] or obese (BMI ≥ 30 kg/m^2^), although this is more prominent for ALT than for AST. After complete viral suppression in patients infected with HBV, ALT elevation indicated high BMI [adjusted odds ratio (OR) 1.78; 95% confidence interval (CI) 1.02–3.11] (10), and ALT levels were significantly higher in chronic HBV infection with MetS (7). In contrast to the De Ritis ratio (AST/ALT ratio > 2.0) for alcoholic hepatitis and >1.0 for cirrhosis/fibrosis (11), and ALT/AST ratio >1 could be independently associated with MetS (12, 13) and fatty liver disease (14, 15). The index might offer more predictive power when considering more confounders, including lifestyle variables and viral hepatitis.

Although aminotransferase can be easily checked during routine examinations, whether the ALT/AST ratio has a predictive impact on MetS in viral hepatitis endemic areas remains unknown. Thus, we aimed to investigate the relationship between the ALT/AST ratio, MetS, and HBV infection in a rural Taiwanese population.

## Methods

### Population and study design

**Figure 1:**
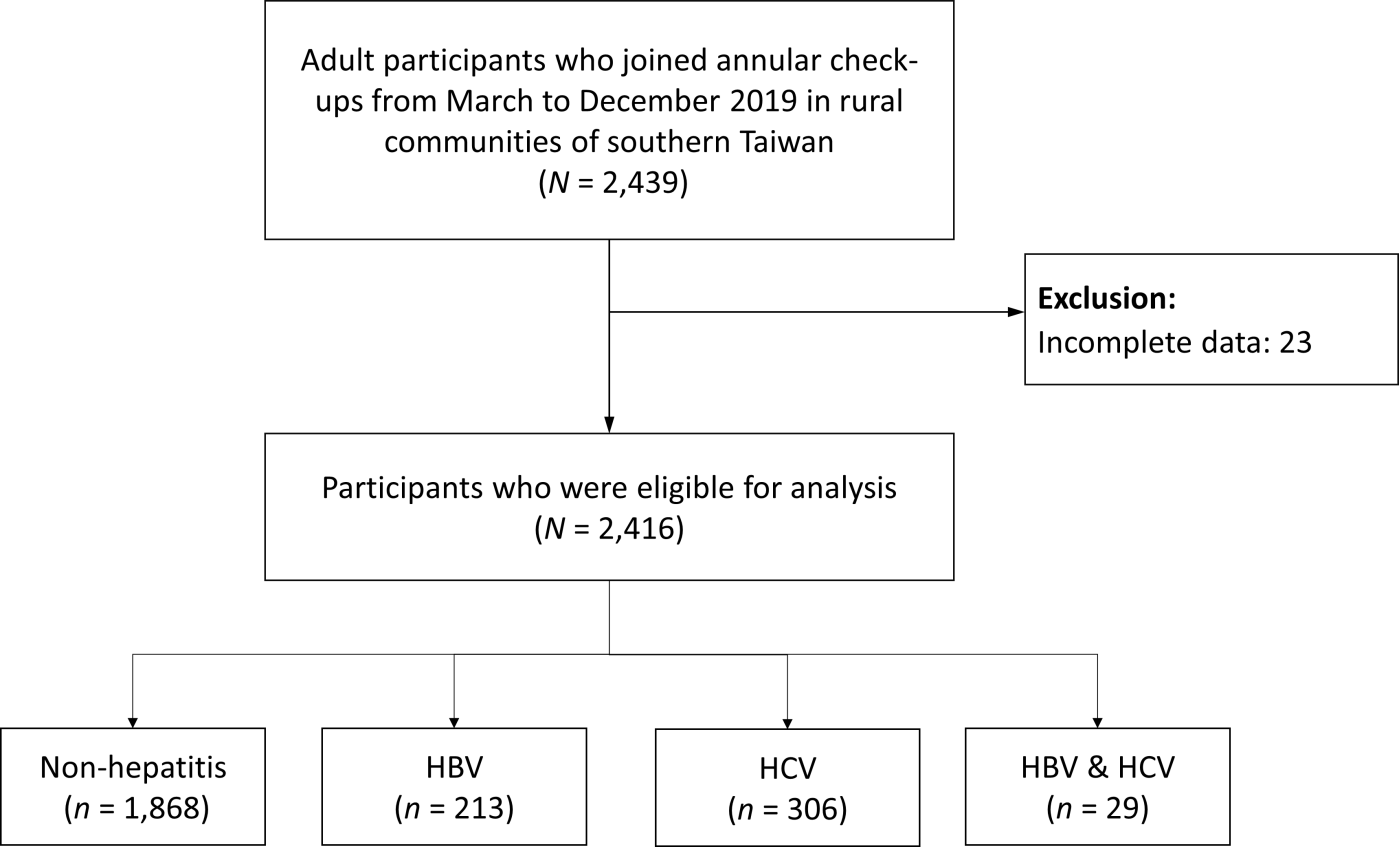
Enrollment of the study participants. HBV, hepatitis B virus; HCV, hepatitis C virus.

This cross-sectional study included adult patients who participated in annual checkups from March to December 2019 in rural communities in southern Taiwan. We collected patient data on personal health habits, laboratory results, and viral markers of hepatitis. All participants signed an informed consent form and completed a questionnaire. After excluding those with incomplete data, 2,416 participants were enrolled in the final analysis (**Figure 1**). This study was approved by the Institutional Review Board and Ethics Committee of Chang Gung Memorial Hospital (IRB No. 201900222A3). According to a previous study reporting the correlation between the presence/absence of MetS and the ALT/AST ratio, the mean (standard deviation) ALT/AST ratio was 1.29 (0.42) and 1.09 (0.41) in men with and without MetS, respectively (16). The mean (standard deviation) ALT/AST ratio was 1.10 (0.35) and 0.87 (0.35) in women with and without MetS, respectively (16). Considering a type I error rate of 1% and power of 99%, a minimum sample size of 418 men and 226 women was required.

### Questionnaire on lifestyle

The questionnaire on lifestyle and demographic characteristics, including sex, age, and educational level (number of years of education received), were included. Participants were asked the following questions regarding three substances and four healthy habits: “Do you smoke cigarettes, chew betel nuts, and regularly consume alcohol or alcohol-related beverages?” Participants were classified as “non-users” if they reported having never smoked, chewed, or drunk and “current/former users” if they reported being current users or previous users who had ceased chewing or smoking. Regarding diet, they were asked the following: “How often do you consume three portions of vegetables (1.5 bowls)? Two portions of fruit (one bowl)? Water intake of at least 1,500 ml per day?” and “How often do you have exercise, for at least >30 min, three times per week?” Responses were categorized as “never,” “seldom,” “usually,” or “always.” For the evaluation, four responses were categorized into two-level frequencies: no—never/seldom and yes—usually/always.

### Anthropometric measurements

Waist circumference was measured using a soft tap and defined at the umbilical level while standing without stress for a moment (17). For blood pressure measurements, all participants underwent two measurements *via* anthropometric equipment in the sitting position after 10 min of rest, and the mean arterial pressure (systolic and diastolic) was recorded (18).

### Biochemistries and serological markers of hepatitis

Blood samples were obtained after fasting for 12 h and tested in the laboratory of the CGMH Hospital. Biochemical tests included serum AST, ALT, TG, LDL-C, HDL-C, TC (Roche Diagnostics, Cobas6000, C501, Germany), and HbA1c (Trinity Biotech, Premier, HB9210, USA). Hepatitis B surface antigen (HBsAg) was detected using routine standard ELISA (General Biological Corp., Hsinchu, Taiwan), and the anti-HCV antibody was evaluated using SP-NANBASE C-96 3.0 plate (General Biological Corp).

### Definition of MetS

MetS was diagnosed based on the modified qualitative criteria of the International Diabetes Federation definition (19), requiring the presence of three or more of the following five criteria: 1) waist circumference >90 cm in men or >80 cm in women for Asians, 2) TG >150 mg/dl, 3) HDL-C <40 mg/dl in men or <50 mg/dl in women, 4) blood pressure >130/85 mmHg or current use of antihypertensive medications, and 5) glycohemoglobin (HbA1c) >5.7 or use of oral antidiabetic agents or insulin.

### Statistical analysis

The demographic characteristics of the participants with different HBV and HCV statuses (none, HBV only, HCV only, and co-infection) were compared using the chi-square test for categorical variables and the independent sample *t*-test for continuous variables. Pairwise comparisons using the Bonferroni correction were performed when the overall test results were significant. We also compared the demographic characteristics between the MetS and non-MetS groups using the chi-square test for categorical variables and the independent sample *t*-test for continuous variables. Using the demographics/characteristics as explanatory variables, a series of univariate logistic regression analyses were performed to initially screen the potentially associated factors of MetS. The multivariable logistic regression model further included variables with significance levels <0.15 (20). Among the indicators of liver function (AST, ALT, and ALT/AST ratio > 1), ALT/AST ratio >1 was selected in the multivariable model to prevent multicollinearity. Finally, a receiver operating characteristic (ROC) curve analysis was conducted to evaluate the ability of the ALT/AST ratio to discriminate the presence of MetS. All tests were two-tailed, and a *p*-value <0.05 was considered significant. Data analyses were performed using SPSS version 25 (IBM SPSS Inc., Chicago, IL, USA).

## Results

### Characteristics of participants

**Table 1** presents the demographics and characteristics of the 2,416 participants who completed the health examination and the questionnaire. The mean age of the participants was 64.1 years [standard deviation (SD) 14.9 years], with 56.1% being >65 years. Women were predominant (63.1%). Nearly one-fourth of the participants (*n* = 548, 22.7%) were seropositive for viral hepatitis, of whom 213 (8.8%) had HBV, 306 (12.7%) had HCV, and 29 (1.2%) were co-infected with HBV and HCV. Approximately 70% of the participants did not exercise regularly, and the distribution difference among all subgroups was insignificant. Compared to HCV patients, those with HBV seropositivity were younger (61.1 ± 12.3 vs. 70.8 ± 10.0 years), had lower education levels (7.7 vs. 3.9 years), and had more alcoholic drinking but less betel nut chewing. Half of the participants (*n* = 1,262, 52.2%) had MetS. The HBV seropositive patients had fewer MetS components and a lower risk of MetS than HCV seropositive patients (41.8% vs. 58.8%). The participants with co-infection had higher ATL and AST levels than the other subgroups (*p *< 0.001), whereas the proportion of patients with an ALT/AST ratio >1 was slightly lower in the HCV subgroup.

**Table 1:** Demographics and characteristics of the study subjects according to the HBV and HCV status (*N* = 2,416).

Variable	Total	None	HBV only	HCV only	HBV and HCV	*p-*value
Number of subjects	2,416	1,868	213	306	29	
Female sex	1,525 (63.1)	1,160 (62.1)	138 (64.8)	210 (68.6)	17 (58.6)	0.149
Age, years	64.1 ± 14.9	63.3 ± 15.6	61.1 ± 12.3	70.8 ± 10.0^a,b^	67.9 ± 10.1	<0.001
Age groups						<0.001
<40 years	214 (8.9)	199 (10.7)	14 (6.6)	0 (0.0)^a,b^	1 (3.4)^c^	
40–64 years	846 (35.0)	653 (35.0)	104 (48.8)^a^	81 (26.5)^a,b^	8 (27.6)	
≥65 years	1,356 (56.1)	1,016 (54.4)	95 (44.6)^a^	225 (73.5)^a,b^	20 (69.0)	
Education level, years	6.5 ± 5.4	6.9 ± 5.5	7.7 ± 5.0	3.9 ± 4.2^a,b^	3.8 ± 4.4^a,b,c^	<0.001
Dietary behavior						
Vegetable intake ≥3 portions per day	1,608 (66.6)	1,279 (68.5)	140 (65.7)	173 (56.5)^a^	16 (55.2)	<0.001
Fruit intake ≥2 portions per day	1,351 (55.9)	1,079 (57.8)	118 (55.4)	140 (45.8)^a^	14 (48.3)	0.001
Water intake ≥1,500 cc per day	1,417 (58.7)	1,139 (61.0)	118 (55.4)	145 (47.4)^a^	15 (51.7)	<0.001
Irregular exercise	1,679 (69.5)	1,295 (69.3)	145 (68.1)	220 (71.9)	19 (65.5)	0.735
Substance use						
Smoking	433 (17.9)	332 (17.8)	37 (17.4)	58 (19.0)	6 (20.7)	0.931
Betel nut chewing	225 (9.3)	164 (8.8)	14 (6.6)	43 (14.1)^a,b^	4 (13.8)	0.010
Alcoholic drinking	243 (10.1)	183 (9.8)	29 (13.6)	29 (9.5)	2 (6.9)	0.314
Data of metabolic syndrome (MetS)						
Waist circumference (WC), cm	84.80 ± 10.8	84.76 ± 10.9	84.3 ± 10.7	85.2 ± 10.0	86.8 ± 10.8	0.564
Systolic blood pressure, mmHg	134.74 ± 20.3	134.66 ± 20.1	131.6 ± 21.0	137.0 ± 21.2^b^	139.3 ± 16.3	0.017
Diastolic blood pressure, mmHg	81.7 ± 12.3	81.8 ± 12.2	82.3 ± 13.1	80.4 ± 12.6	81.5 ± 11.9	0.270
High-density lipoprotein, mg/dl	51.0 ± 13.2	51.2 ± 13.0	52.9 ± 13.5	49.1 ± 13.9^b^	51.0 ± 15.4	0.011
Glycosylated hemoglobin, mg/dl	6.09 ± 1.07	6.08 ± 1.04	6.00 ± 1.03	6.26 ± 1.25^a,b^	6.10 ± 0.87	0.028
Triglyceride, mg/dl	137.6 ± 95.9	140.1 ± 98.7	117.8 ± 82.1^a^	137.4 ± 87.5	121.4 ± 73.1	0.011
Metabolic syndrome (MetS)	1,262 (52.2)	973 (52.1)	89 (41.8)^a^	180 (58.8)^b^	20 (69.0)^b^	<0.001
Liver and renal function						
AST, U/L	25.9 ± 13.8	24.8 ± 11.6	26.9 ± 11.2	29.8 ± 21.3^a^	46.1 ± 32.7^a,b,c^	<0.001
ALT, U/L	24.2 ± 19.1	23.4 ± 18.6	25.9 ± 17.7	26.1 ± 21.2	41.2 ± 29.7^a,b,c^	<0.001
ALT/AST ratio	0.90 ± 0.34	0.91 ± 0.34	0.94 ± 0.34	0.86 ± 0.29^a,b^	0.92 ± 0.26	0.036
ALT/AST >1	666 (27.6)	525 (28.1)	61 (28.6)	69 (22.5)	11 (37.9)	0.121

Data were presented as mean ± standard deviation or frequency and percentage. “a,” “b,” and “c” indicate significant differences versus the “None”, “HBV only,” and “HCV only” groups in the Bonferroni multiple comparison, respectively.

HBV, hepatitis B virus; HCV, hepatitis C virus; BMI, body mass index; AST, aspartate aminotransferase; ALT, alanine aminotransferase; eGFR, estimated glomerular filtration rate.

### Characteristics of MetS

**Table 2** presents the demographics and characteristics of participants with and without MetS. Compared to the patients without MetS, those with MetS were older (67.0 ± 12.8 vs. 61.0 ± 16.4 years, *p *< 0.001), had lower education levels (5.5 ± 5.1 vs. 7.7 ± 5.6 years, *p *< 0.001), had less fruit intake at ≥2 portions per day (53.2% vs. 58.9%, *p* = 0.004), were more likely to do betel nut chewing (11.1% vs. 7.4%, *p* = 0.002), had significantly positive data of individual MetS components, and had higher AST (27.4 ± 15.8 vs. 24.2 ± 11.0, *p *< 0.001) and ALT levels (27.1 ± 21.0 vs. 21.0 ± 16.2 U/L, *p* < 0.001). The MetS group had a significantly higher ALT/AST ratio >1 (34.2% vs. 20.4%, *p* < 0.001) and higher proportion of HCV seropositivity (14.3% vs. 10.9%) than the non-MetS group. Meanwhile, the prevalence of HBV seropositivity was higher in the non-MetS group than in the MetS group (10.7% vs. 7.1%).

**Table 2:** Demographics and characteristics of the study subjects according to the status of MetS (*N* = 2,416).

Variable	MetS	Non-MetS	*p*
Number of subjects	1,262	1,154	
Female sex	803 (63.6)	722 (62.6)	0.588
Age, years	67.0 ± 12.8	61.0 ± 16.4	<0.001
Age groups			<0.001
<40 years	51 (4.0)	163 (14.1)	
40–64 years	408 (32.3)	438 (38.0)	
≥65 years	803 (63.6)	553 (47.9)	
Education level, years	5.5 ± 5.1	7.7 ± 5.6	<0.001
Dietary behavior			
Vegetable intake ≥3 portions per day	820 (65.0)	788 (68.3)	0.085
Fruit intake ≥2 portions per day	671 (53.2)	680 (58.9)	0.004
Water intake ≥1,500 cc per day	743 (58.9)	674 (58.4)	0.815
Irregular exercise	899 (71.2)	780 (67.6)	0.052
Substance use			
Smoking	236 (18.7)	197 (17.1)	0.297
Betel nut chewing	140 (11.1)	85 (7.4)	0.002
Alcoholic drinking	129 (10.2)	114 (9.9)	0.779
Data of metabolic syndrome (MetS)			
Waist circumference (WC), cm	89.9 ± 9.3	79.2 ± 9.4	<0.001
Systolic blood pressure, mmHg	141.5 ± 19.0	127.4 ± 19.1	<0.001
Diastolic blood pressure, mmHg	84.4 ± 12.4	78.6 ± 11.5	<0.001
High-density lipoprotein, mg/dl	45.5 ± 11.5	57.2 ± 12.2	<0.001
Glycosylated hemoglobin, mg/dl	6.4 ± 1.2	5.7 ± 0.78	<0.001
Triglyceride, mg/dl	175.0 ± 111.1	96.6 ± 50.4	<0.001
Liver and renal function			
AST, U/L	27.4 ± 15.8	24.2 ± 11.0	<0.001
ALT, U/L	27.1 ± 21.0	21.0 ± 16.2	<0.001
ALT/AST >1	431 (34.2)	235 (20.4)	<0.001
HBV and HCV status			<0.001
None	973 (77.1)	895 (77.6)	
HBV only	89 (7.1)	124 (10.7)	
HCV only	180 (14.3)	126 (10.9)	
HBV and HCV	20 (1.6)	9 (0.78)	

Data were presented as mean ± standard deviation or frequency and percentage.

MetS, metabolic syndrome; AST, aspartate aminotransferase; ALT, alanine aminotransferase; eGFR, estimated glomerular filtration rate; HBV, hepatitis B virus; HCV, hepatitis C virus.

### MetS-associated risk factors

The univariate logistic regression analyses revealed that the following covariates might be associated with MetS: age, education level, fruit intake, betel nut chewing, AST level, ALT level, ALT/AST ratio >1, and seropositivity to HBV and HCV (**Table 3**). After incorporating the variables whose significant levels were <0.15 in the univariate analyses, the multivariable model identified that older age (OR 1.02, 95% CI 1.01–1.03) and the presence of ALT/AST ratio >1 (OR 2.63, 95% CI 2.15–3.21) were significantly associated with a greater risk of MetS. By contrast, a high education level (OR 0.96, 95% CI 0.94–0.98) and HBV seropositivity (OR 0.70, 95% CI 0.52–0.95) were also significantly inversely related to the risk of MetS. Additionally, betel nut chewing was associated with a higher risk of MetS (OR 1.41, 95% CI 1.05–1.90).

**Table 3:** Association between demographics/characteristics and the risk of metabolic syndrome (*N* = 2,416).

	Univariate analysis		Multivariable analysis^a^	
Variable	OR (95% CI)	*p*	OR (95% CI)	*p*
Female sex	1.05 (0.89–1.24)	0.588		
Age, years	1.028 (1.022–1.034)	<0.001	1.02 (1.01–1.03)	<0.001
Education level, years	0.93 (0.91–0.94)	<0.001	0.96 (0.94–0.98)	<0.001
Vegetable intake ≥3 portions per day	0.86 (0.73–1.02)	0.085	1.19 (0.92–1.55)	0.189
Fruit intake ≥2 portions per day	0.79 (0.67–0.93)	0.004	0.82 (0.63–1.05)	0.114
Water intake ≥1,500 cc per day	1.02 (0.87–1.20)	0.815		
Irregular exercise	1.187 (0.998–1.412)	0.052	1.12 (0.93–1.35)	0.227
Smoking	1.12 (0.91–1.38)	0.297		
Betel nut chewing	1.57 (1.18–2.08)	0.002	1.41 (1.05–1.90)	0.021
Alcoholic drinking	1.04 (0.80–1.35)	0.779		
AST, U/L	1.02 (1.01–1.03)	<0.001		
ALT, U/L	1.022 (1.016–1.028)	<0.001		
ALT/AST >1^b^	2.03 (1.69–2.44)	<0.001	2.63 (2.15–3.21)	<0.001
HBV and HCV status				
None	Reference		Reference	
HBV only	0.66 (0.50–0.88)	0.005	0.70 (0.52–0.95)	0.021
HCV only	1.314 (1.028–1.679)	0.029	1.03 (0.79–1.33)	0.836
HBV and HCV	2.04 (0.93–4.51)	0.077	1.52 (0.67–3.43)	0.312

OR, odds ratio; CI, confidence interval; BMI, body mass index; AST, aspartate aminotransferase; ALT, alanine aminotransferase; eGFR, estimated glomerular filtration rate; HBV, hepatitis B virus; HCV, hepatitis C virus.

a Those variables whose significant levels were less than 0.15 were further included in the multivariable logistic regression model.

b Among the indicators of liver function (AST, ALT, ALT/AST ratio, and ALT/AST ratio >1), ALT/AST ratio >1 was chosen in the multivariable model to prevent the problem of multicollinearity.

### Using the ALT/AST ratio to discriminate MetS

Due to the presence of an ALT/AST ratio >1 as an associated factor for MetS, we assessed its ability to discriminate the presence of MetS. The results revealed a modest discrimination performance with an area under the ROC curve of 61.8% (95% CI 59.5%–64.0%). The derived optimal cutoff determined by the Youden index was >0.852, with a sensitivity of 56.7% (95% CI 53.9%–59.4%) and a specificity of 62% (95% CI 59.1%–64.8%) (data not shown).

## Discussion

This community-based participatory research investigates MetS-related factors, including diet, exercise, and education, and uses the ALT/AST ratio as a predictive index for MetS. Our findings suggest that an ALT/AST ratio >1 might increase the risk of MetS (OR 2.63), whereas high education and HBV seropositivity are inversely associated with MetS. The effects of the ALT/AST ratio and HBV seropositivity on MetS prediction differed. High ALT levels were significantly associated with MetS in women (21) and reflected central obesity with advanced steatohepatitis. In this study, an ALT/AST ratio >1 could be a simple index to predict MetS by considering all dietary content, healthy behaviors, and education, especially in viral hepatitis endemic areas. The diverse effects of the ALT/AST ratio and HBV seropositivity might exhibit unlinked pathophysiologies, such as hepatosteatosis or hepatic fibrosis in MetS.

Although serum viral load, AST, and ALT levels were independent predictors of histological grade (22), a single ALT or AST test could not offer a strong association between MetS and abnormal liver function, especially in patients with viral hepatitis. Chen et al. have also reported that men had a three times higher risk of MetS than women, who had fewer metabolic abnormalities and elevated ALT levels (12). The ALT/AST ratio is straightforward and feasible for use in community health examinations. The index can be presented as either hepatic fibrosis or steatosis in different studies (13–15). A previous study has reported that a higher ALT/AST ratio is associated with insulin resistance in metabolically unhealthy Korean individuals (13). Moreover, ALT/AST ratio >1 was significantly associated with MetS in the Thai population (16); however, variables correlated with lifestyle and viral hepatitis were absent. Here, the ALT/AST ratio was independently associated with MetS. Zhao et al. have reported that the ALT/AST ratio could predict insulin resistance and MetS among the Chinese population (23), although this was not the case in our study, which had a high proportion of viral hepatitis. A high ALT/AST ratio is associated with fatty liver, a significant component of MetS, hypertriglyceridemia, and steatohepatitis.

Our findings further demonstrate that HBV infection is inversely associated with MetS, which is consistent with the findings of previous studies. Significant hepatic impairment in co-infection with HBV/HCV was observed, but patients with HBV had minor liver dysfunction, were younger, and had a higher education level (7.7 ± 5.0), healthy dietary behavior, and less betelnut chewing. Kuo et al. have reported that HBV infection was inversely associated with MetS only in lean patients (*p* = 0.002) but not in the general population (1). Joo et al. have reported that HBsAg seropositivity in Korean adults was associated with a lower risk of developing non-alcoholic fatty liver disease (NAFLD), indicating a possible effect of HBV infection on the pathogenesis of NAFLD in a cohort study (24). A body of evidence has also indicated that patients with chronic hepatitis B (CHB) have a lower incidence of NAFLD and steatohepatitis. A possible mechanism is that HBV viral activity might protect against hepatic steatosis and metabolic disturbances. The severity of steatosis was inversely associated with HBV viral load (25, 26). In an animal model, steatosis inhibited HBV replication by reducing HBV DNA and HBV-related antigens (27).

Nevertheless, patients with CHB with coexisting components of MetS are associated with more severe liver diseases. Li et al. have reported that fatty liver was significantly associated with higher HBsAg seroclearance in patients with CHB (28), while concurrent NAFLD might inhibit HBV replication and promote HBsAg seroclearance (29). However, the fatty liver also exacerbates liver fibrosis. Khalili et al. have reported that MetS was prevalent in this HBV group and independently associated with higher ALT levels (7). Moreover, Cai et al. have reported that HBV comorbidity with fibrosis increases the MetS component burden (30), and Yan et al. discovered that cirrhosis is prevalent in HBV with MetS (4.83% vs. 2.93%, compared with non-MetS; *p* = 0.002) (6). Additionally, Chan et al. revealed that overweight and concurrent fatty liver disease are associated with increased mortality risk and hepatocellular carcinoma in patients with CHB (31). Chien et al. have reported that patients who were unaware of their hepatitis B infection tended to have a higher risk of central obesity, hyperglycemia, insulin resistance, and MetS than those who were aware of their hepatitis B infection (OR 1.85, *p *< 0.05). In patients without MetS, HBV with MetS has a higher ALT level and ALT/AST ratio, suggesting a prominent hepatic inflammation and a predictor of steatohepatitis.

In addition to health promotion, regular physical exercise, cessation of alcohol or betelnut consumption, good dietary habits, aggressive follow-up, and early detection using the ALT/AST ratio may reduce MetS or steatohepatitis burden in chronic HBV infection/carriers.

### Limitations

This study had several inherent limitations in its cross-sectional design. First, details of the hepatobiliary disease, fatty liver, antiviral therapeutic response, and viral load/activity were unavailable in the research. Nevertheless, the early implementation of HBV vaccination may influence personal lifestyles, behaviors, and insight for disease screening. Second, we lacked the sequential results of aminotransferase levels and detected all the factors influencing liver function. Although the ALT/AST ratio could not reflect an accurate level, the index is a different part of hepatic injury. Third, we could not obtain details of personal health conditions and medications associated with hepatic function. Finally, many non-invasive waist-to-height ratios or echography approaches could be used for predicting MetS (32, 33); however, interoperator variability and infeasible devices in community checkups limit their clinical application.

## Conclusion

An ALT/AST ratio >1 is independently associated with MetS after adjusting for age, lifestyle, education level, and viral hepatitis seropositivity. Although HBV seropositivity and higher education are inversely associated with MetS, the ALT/AST ratio remains a reliable predictor of MetS and a simple index for community checkups. Nevertheless, the corresponding biological mechanisms of HBV in MetS remain to be elucidated, and future large-scale studies are needed to survey this association.

## Data availability statement

The original contributions presented in the study are included in the article/supplementary material. Further inquiries can be directed to the corresponding author/s.

## Ethics statement

This study was reviewed and approved by The Institutional Review Board approved the study (IRB NO: 201900222A3). Written informed consent was obtained from individual or guardian participants. The patients/participants provided their written informed consent to participate in this study.

## Author contributions

All authors contributed to the statistical analysis and writing of the study. M-SL, H-SL, and M-YC participated in the study design, data acquisition, and critical review and wrote the manuscript. M-LC, M-HT, and Y-YH participated in the analysis and interpretation of data. Y-SL, M-ST, and C-LY collected the data and contributed to the study direction. All authors contributed to the article and approved the submitted version.

## Funding

The study was supported by a grant from Taiwan Formosa Plastic Group (FCRPF6L0011). The authors thank Mr. Alfred Hsing-Fen Lin, M.S., and Mrs. Bing-Yu Chen, Ph.D., for their statistical analysis.

## Conflict of interest

The authors declare that the research was conducted in the absence of any commercial or financial relationships that could be construed as a potential conflict of interest.

## Publisher’s note

All claims expressed in this article are solely those of the authors and do not necessarily represent those of their affiliated organizations, or those of the publisher, the editors and the reviewers. Any product that may be evaluated in this article, or claim that may be made by its manufacturer, is not guaranteed or endorsed by the publisher.
